# Investigation on Characteristic Variation of the FBG Spectrum with Crack Propagation in Aluminum Plate Structures

**DOI:** 10.3390/ma10060588

**Published:** 2017-05-27

**Authors:** Bo Jin, Weifang Zhang, Meng Zhang, Feifei Ren, Wei Dai, Yanrong Wang

**Affiliations:** 1 School of Energy and Power Engineering, Beihang University, 37 Xueyuan Rd., Haidian Dist., Beijing 100191, China; by1504121@buaa.edu.cn (B.J.); yrwang@buaa.edu.cn (Y.W.); 2 School of Reliability and Systems Engineering, Beihang University, 37 Xueyuan Rd., Haidian Dist., Beijing 100191, China; 08590@buaa.edu.cn (W.Z.); zhangmeng123@buaa.edu.cn (M.Z.); RenFeiFei@buaa.edu.cn (F.R.); 3 Collaborative Innovation Centre for Advanced Aero-Engine, Beihang University, 37 Xueyuan Rd., Haidian Dist., Beijing 100191, China

**Keywords:** FBG sensor, reflection intensity spectra, *T*-matrix method

## Abstract

In order to monitor the crack tip propagation of aluminum alloy, this study investigates the variation of the spectrum characteristics of a fiber Bragg grating (FBG), combined with an analysis of the spectrum simulation. The results identify the location of the subordinate peak as significantly associated with the strain distribution along the grating, corresponding to the different plastic zones ahead of the crack tip with various crack lengths. FBG sensors could observe monotonic and cyclic plastic zones ahead of the crack tip, with the quadratic strain distribution along the grating at the crack tip-FBG distance of 1.2 and 0.7 mm, respectively. FBG sensors could examine the process zones ahead of the crack tip with the cubic strain distribution along the grating at the crack tip-FBG distance of 0.5 mm. The spectrum oscillation occurs as the crack approaches the FBG where the highly heterogeneous strain is distributed. Another idea is to use a finite element method (FEM), together with a *T*-matrix method, to analyze the reflection intensity spectra of FBG sensors for various crack sizes. The described crack propagation detection system may apply in structural health monitoring.

## 1. Introduction

Aluminum alloys are widely utilized in aircraft structures, and the aircraft will suffer from known structural damage in long-time service, including fatigue, corrosion, material aging, and cracking problems, etc. [1]. Moreover, the structural damage can be detected in a timely manner with real-time monitoring of the aluminum alloy crack propagation [2]. Fiber Bragg grating (FBG) sensors have been recognized and applied in structural health monitoring (SHM), such as in the concrete structure of large dams, nuclear power stations, offshore platforms, and so on [3], due to the following distinguishing advantages of FBG sensors: small size, corrosion resistance, electromagnetic resistance, multiplexing, etc. [4]. It is considered to be one of the most promising sensors in monitoring the stress-strain distribution around the crack tip for the prognosis of crack propagation [5]. However, it is challenging to use the FBG sensors in crack propagation monitoring of aluminum alloy components.

Several researchers have made contributions on characteristics extracted from crack propagation in aluminum alloy structures with FBG sensors. Yuan [6] extracted the crack initiation factor (CIF) and propagation factor (CPF) from the frequency analysis of the FBG spectrum with wavelet analysis. Opoka [7] extracted the damage parameter as a damage indicator by a root mean square deviation (RMSD) estimator and the smoothed frequency spectrum of the strain data. Peters [8] discussed the spectra of the FBG as a possible basis for the resolution of an arbitrary applied strain distribution. However, it lacked physical interpretations of their evaluation results.

In health monitoring of composite materials, some research has been conducted on the extracted physical characteristics. Nobuo [9] proposed two peaks at shorter and longer wavelengths in the reflection spectrum, corresponding to the strain level of the boned and delaminated area. Takeda [10] found that the spectrum has two peaks depending on the strain distribution caused by the edge delamination, and the intensity of the higher wavelength peak increased with the extent of the edge delamination. Such a spectrum change against the delamination growth was also reported in his previous research [11]. The reflection spectrum from the FBG sensors was very sensitive to the delamination growth, and the intensity ratio of the two peaks in the spectrum was proposed as an indicator for the prediction of the delamination size [12].

However, no literature discussing the subordinate peak of the FBG sensors applied in crack monitoring of aluminum alloy has been reported. In this paper, we demonstrated various locations of the subordinate peak, showing a strong correlation to the plastic zone with the crack propagation.

## 2. Experiment

In order to investigate the physical parameters of the spectral intensity varying with the crack lengths, in particular, the variety of the subordinate peaks are used as a quantitative measurement indicator of the crack length, and consequently, the aluminum alloy structural component is equipped with FBG sensors, so that the state and physical parameters can be tracked with the crack propagation.

### 2.1. Specimen

**Figure 1:**
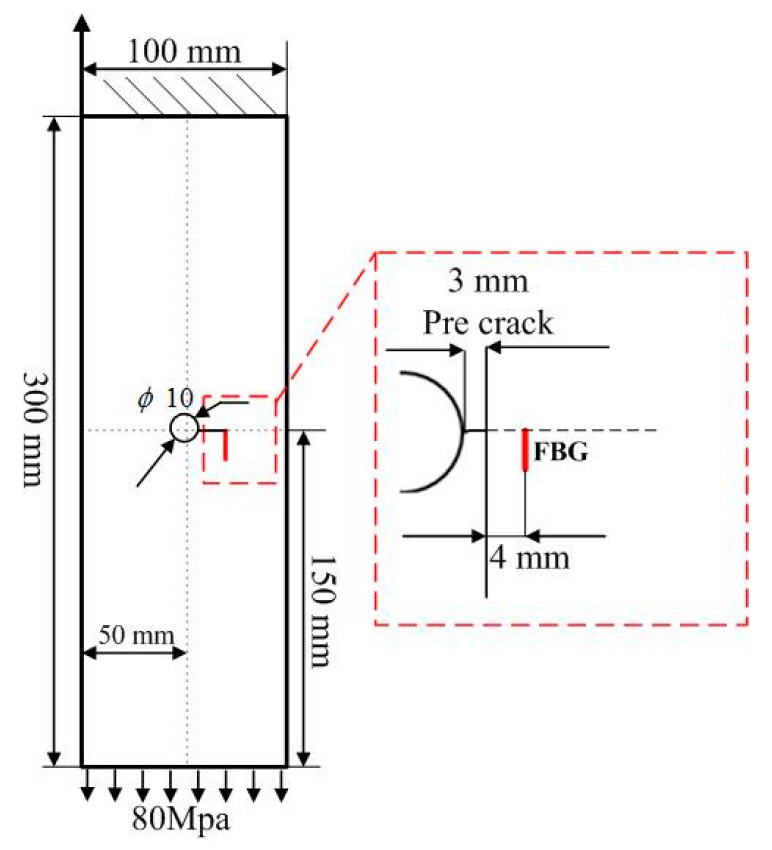
Schematic of the aluminum specimen.

The target system studied in this paper is a plate made of 7075-T6 aluminum alloy, with dimensions of 300 mm × 100 mm × 2 mm, as shown in Figure 1. The properties of the specimen are presented in Table 1. An 8 mm hole is in the center of the plate. A 3 mm pre-crack is introduced by electric discharge machining to trigger fatigue crack propagation. The top boundary is fixed and a uniform tensile loading of 80 MPa is applied to the bottom, as shown in Figure 1. The FBG sensor is glued uniformly onto the specimen surface using liquid cyanoacrylate adhesive, and the adhesive Young’s modulus is 1.7 MPa. The length of the FBG sensor is 10.1 mm. The horizontal distances from the FBG to the hole edge is 7 mm. In order to sense the variation of the axial strain profile in the crack growth, the terminal of the FBG is on the extending line of the crack tip.

### 2.2. Experiment Setup

**Figure 2:**
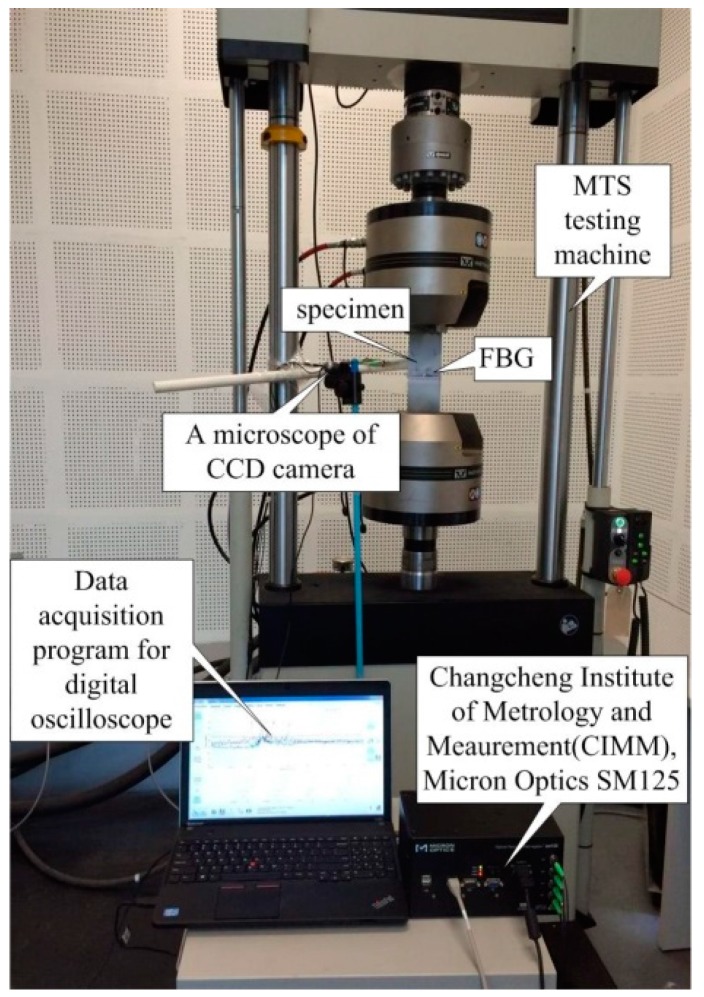
Experiment setup for the FBG sensor damage detection system.

An experiment platform for fatigue crack damage detection is developed and the FBG sensor is utilized to extract the damage characteristics. The experimental setup of the hole-edge crack detection is composed of three major parts: an optical sensing system, a fatigue crack measurement system, and a fatigue load-cycling system, as shown in Figure 2. The reflection intensity spectra of the FBG sensors (model number FSSR5025, Changcheng Institute of Metrology and Measurement, Beijing, China) is obtained by an optical demodulator with high wavelength accuracy (SM125, Micro Optics Inc., Danbury, CT, USA). Fatigue testing is undertaken using a hydraulic MTS machine with constant fatigue loading along the z-direction in Figure 1. A traveling optical microscope monitors the fatigue crack growth, with a charge coupled device (CCD) camera during the loading process. The constant amplitude loading cycling is set at 80 MPa as the maximum value and a frequency of 5 Hz.

## 3. Results

The natural crack of the specimens was initiated and propagated by submitting the specimens to the cyclic fatigue test, and monitoring data were acquired periodically throughout the cyclic fatigue using the FBG sensing demodulation system described previously.

**Figure 3:**
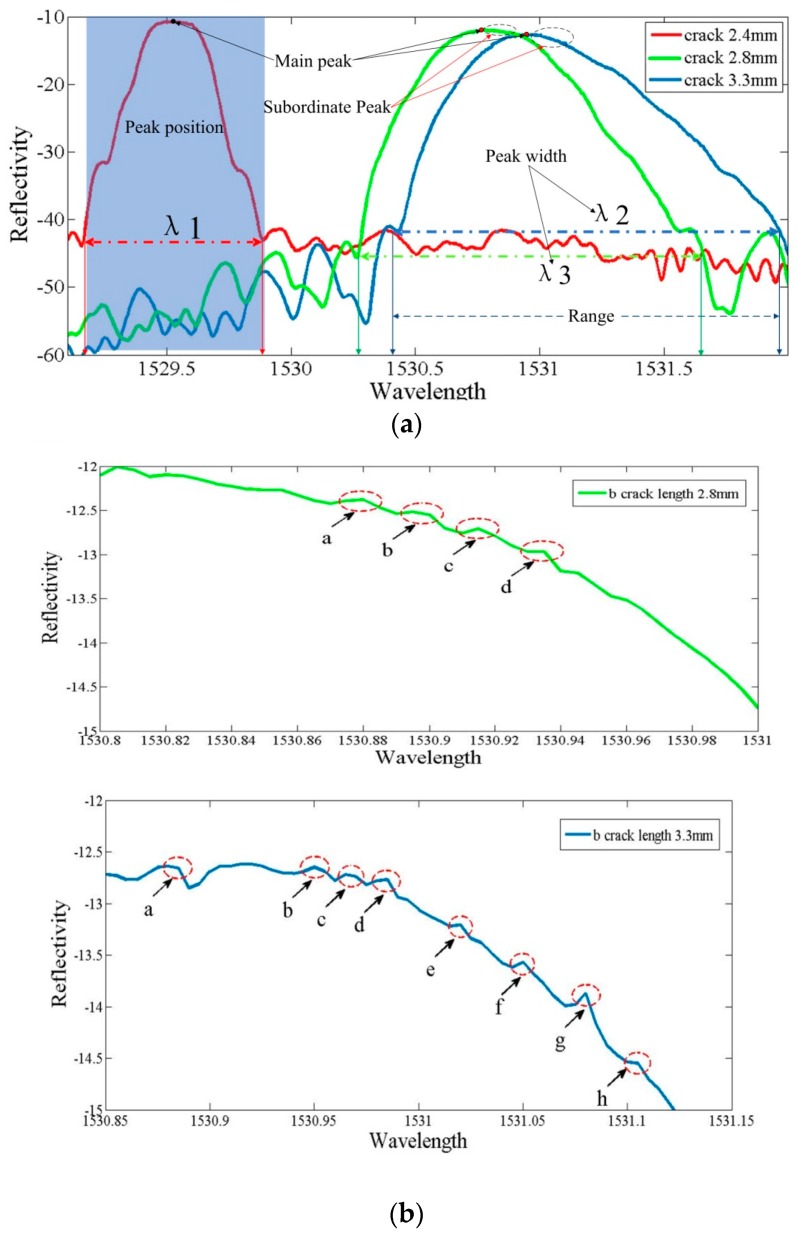
Variety of physical parameters with different crack lengths. (**a**) The global curve at different crack lengths; and (**b**) the subordinate peaks with a 3 db decline near the main peak.

When the sensor is subjected to uniform changes in strain, all grating periods experience changes synchronously, resulting in a shift of the Bragg wavelength without modification of the spectrum shape and, consequently, the spectrum was symmetric and smooth due to the uniformity of the strain distribution along the grating, as the red curve in Figure 3 shows. Furthermore, Figure 3 shows the spectral intensity shift toward a higher wavelength with a decrease of the crack tip-FBG distance from 1.6 mm to 1.2 mm, which indicates an increase of tensile strain in the grating. Thus, the central wavelength enlarges from 1529.5 nm to 1530.7 nm, and the spectrum width broadened from 0.3 nm to 0.8 nm.

It is worth noting that the spectrum persistently enlarges at the crack tip-FBG distance of 1.2 mm, which indicates a chirp, and the region of the subordinate peaks appeared in the higher wavelength between 1530.9 nm and 1531.1 nm, without subordinate peaks appearing in the lower wavelength, as the blue curve in Figure 3a shows.

It is well known that the deformation of the reflected FBG spectrum is generally subjected to the strain gradient distribution along the FBG sensors [13,14]. In fact, the strain gradient ahead of the crack tip along the grating corresponded with the crack tip-FBG distance. While a broadening is anticipated due to the strain field, which changes along the grating, leading to a chirp, one would also expect an increase in wavelength. Thus, the number of the subordinate peaks increased, the spectrum wavelength enlarged from 1530.7 nm to 1530.9 nm, and the spectrum width broadened from 0.8 nm to 1.2 nm. In addition, the subordinate peaks are more obvious, which are located at a higher wavelength, illustrated by comparing the blue and green curves in Figure 3b.

**Figure 4:**
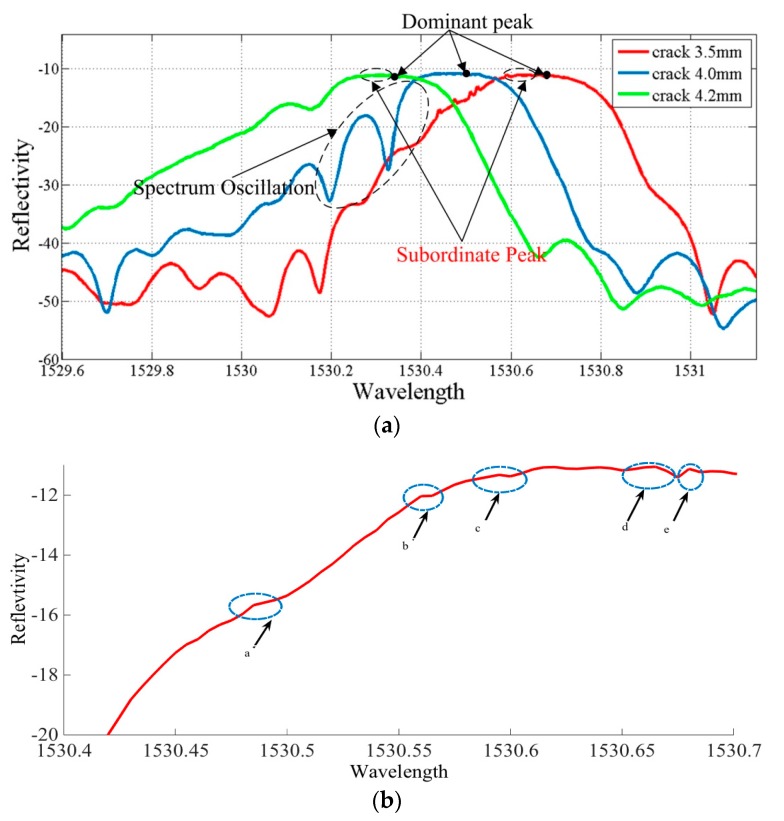
Variety of physical parameters with different crack lengths. (**a**) The global curve at different crack lengths; and (**b**) the subordinate peaks with a 3 db decline near the main peak.

The subordinate peak, as the red curve in Figure 4a has shown, initially appeared in the lower wavelength at the crack tip-FBG distance of 0.5 mm, and the local amplification of the subordinate peak is shown in Figure 4b. The primary grating sensed the uniformity strain without the complex plastic deformation ahead of the crack tip. Thus, the dominant wavelength decreased from 1530.7 nm to 1530.3 nm. However, the grating terminal, approaching the crack tip zone, could sense complex plastic deformation in this area. This means that the grating period chirped at the initial part of the grating. Literature [15] indicates that the spectrum deformation in this case might be the result of a change in the optical properties of the FBG.

The crack propagates perpendicular to the grating under cyclic loading, and when the crack approaches the observation point (FBG), a highly non-uniform strain field is applied along the initial part of the grating; thus, the spectrum oscillation appeared at a crack length of 4.0 mm with a clear subordinate peak, and the spectra are not symmetrical, as the blue curve in Figure 4a illustrates.

In particular, the strain variation in the grating region modifies the grating period and effective index of the fiber [16], thus resulting in a modification of the Bragg condition. In addition, an effective grating period $\Lambda_{eff}$, which includes a single parameter, changed with the physical variation of the grating pitch in the deformed state, and there is a local small perturbation of the index of refraction. More importantly, the number of the subordinate peaks declined with the crack propagation across the FBG at 0.2 mm.

## 4. Discussion

### 4.1. Analysis of Strain Distribution Ahead of the Crack Tip

The strain values along the grating were extracted with the different crack lengths by the finite element method (FEM) during simulation. Based on the strain distribution along the grating, the finite element method was used for the spectrum calculation.

**Figure 5:**
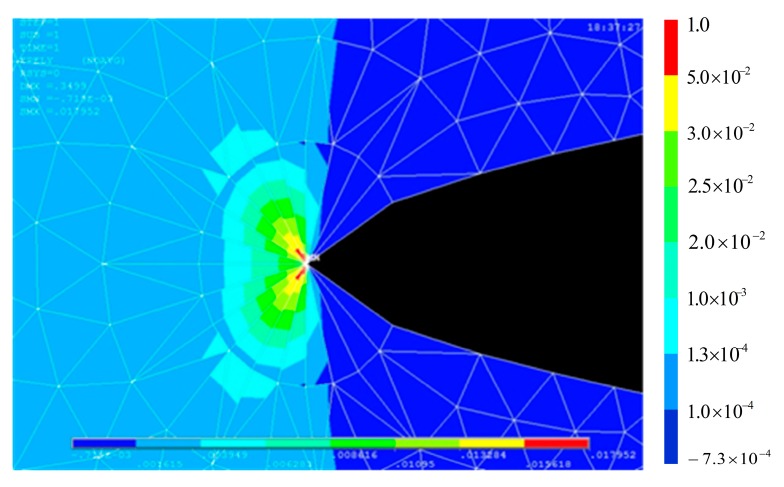
Meshes around the crack.

At the crack tip, the singular element was used to improve the accuracy of the strain distribution around the crack tip, and C3D8R eight-node linear hexahedral elements were used for the other grid. The substrate mesh size was 2 mm, and in order to ensure the accuracy of the calculation, the stress concentration parts were refined, and the mesh size of the crack is 0.5 mm, as shown in Figure 5.

**Figure 6:**
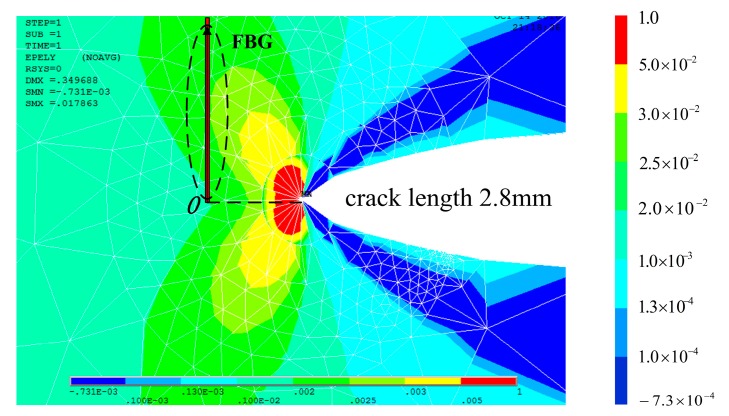
Finite element analysis of a crack with a length of 2.8 mm.

**Figure 7:**
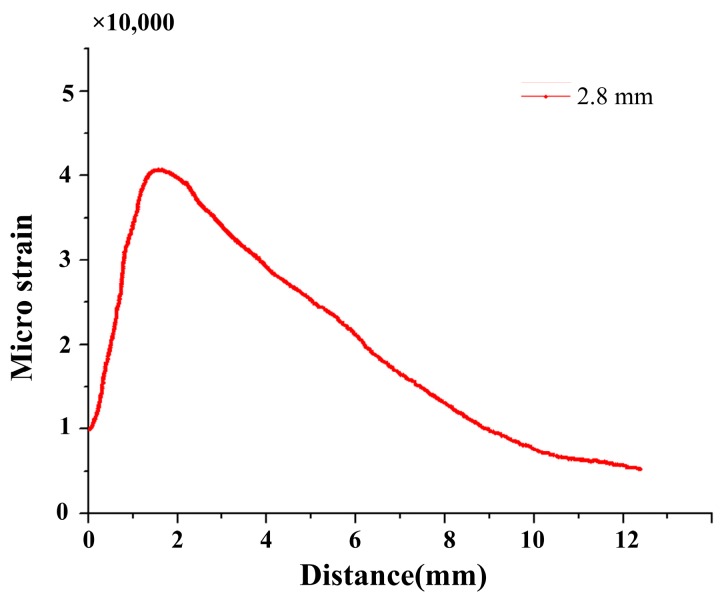
The quadratic distribution along the axis of the FBG.

The FEM is employed to compute the strain distribution along the grating for various crack sizes, such as 2.8 mm, 3.3 mm, 3.5 mm, and 4.0 mm. Figure 6 shows that the monotonic plastic zone ahead of the crack tip is sensed by the sub-region of the grating at the crack tip-FBG distance of 1.2 mm. The strain distribution along the axis of the grating was mainly quadratic, as shown in Figure 7.

**Figure 8:**
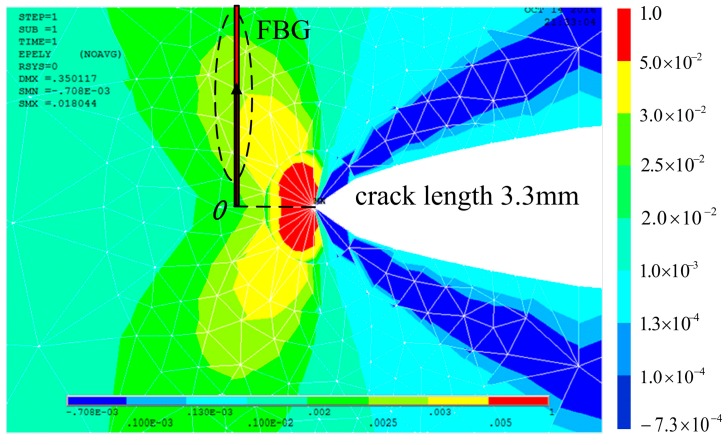
Finite element analysis of a crack with a length of 3.3 mm.

**Figure 9:**
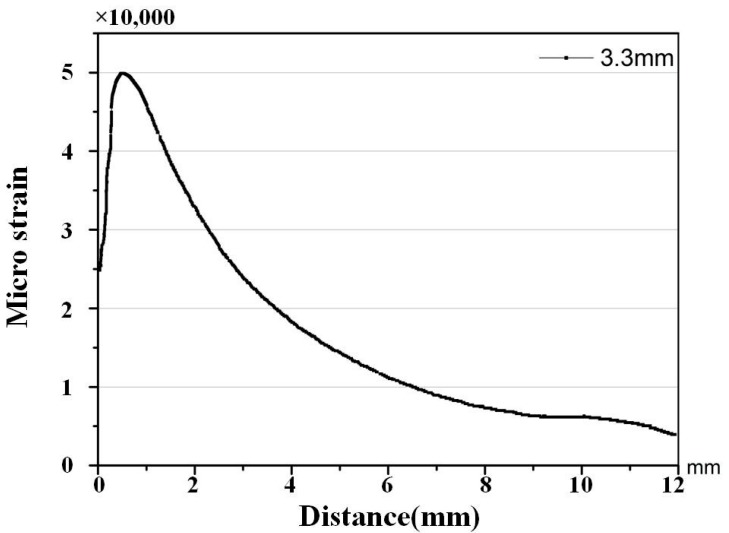
The quadratic distribution along the axis of the FBG.

Figure 8 shows the cyclic plastic zone at the crack tip accesses the sub-region of the grating at the crack tip-FBG distance of 0.7 mm. Heterogeneous strain distribution in this area is more severe than the monotonic plastic zone, which is mainly quadratic, as shown in Figure 9. This is the extent of the subordinate peak that is observable.

**Figure 10:**
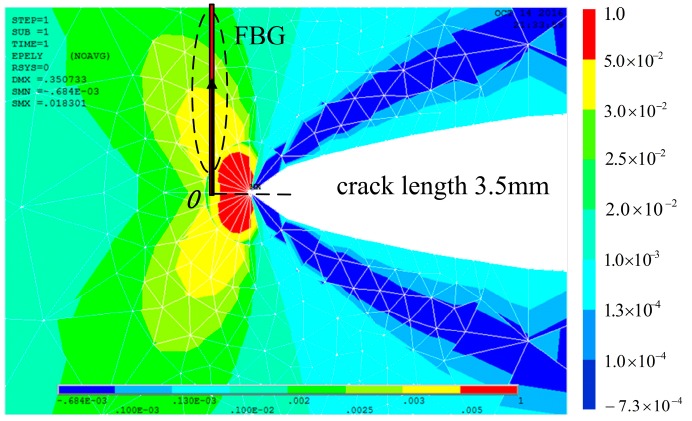
Finite element analysis of a crack with a length of 3.5 mm.

**Figure 11:**
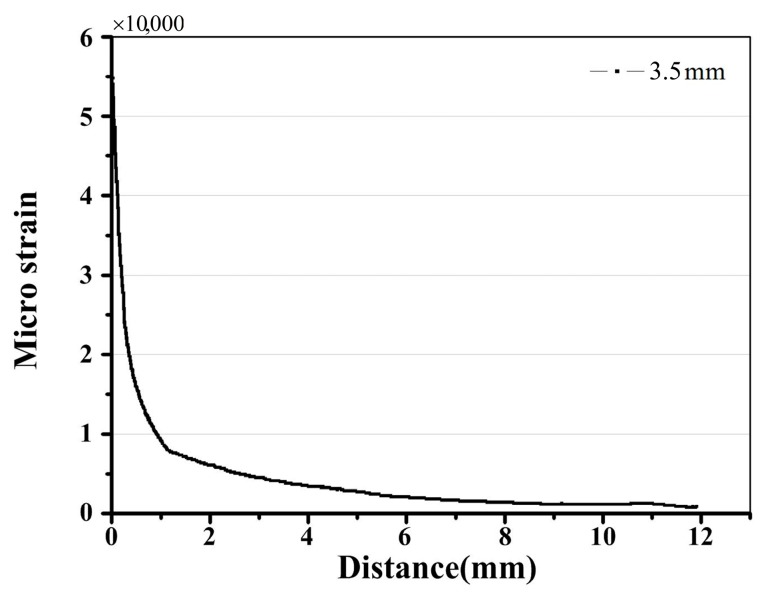
The cubic distribution along the axis of the FBG.

The process plastic zone is accessible to the grating at a crack tip-FBG distance of 0.5 mm. Since the extent of the heterogeneous strain distribution increased with the decrease of the crack tip-FBG distance, the location of subordinate peaks appeared in the lower wavelength, as shown in Figure 10 and Figure 11. Even the spectral oscillations appeared when the crack reaches the FBG (crack length 4.0 mm). A detailed explanation of the spectra phenomenon is discussed in Section 4.4.

### 4.2. Analysis of the FBG Spectrum

The simulation data from FEM and *T*-matrix methods were used to reconstruct the reflection intensity spectrum. These spectra are used for the inversion of the property parameters of the FBG sensors based on the *T*-matrix method [17]. Properties of FBG sensors are listed in Table 2.

**Figure 12:**
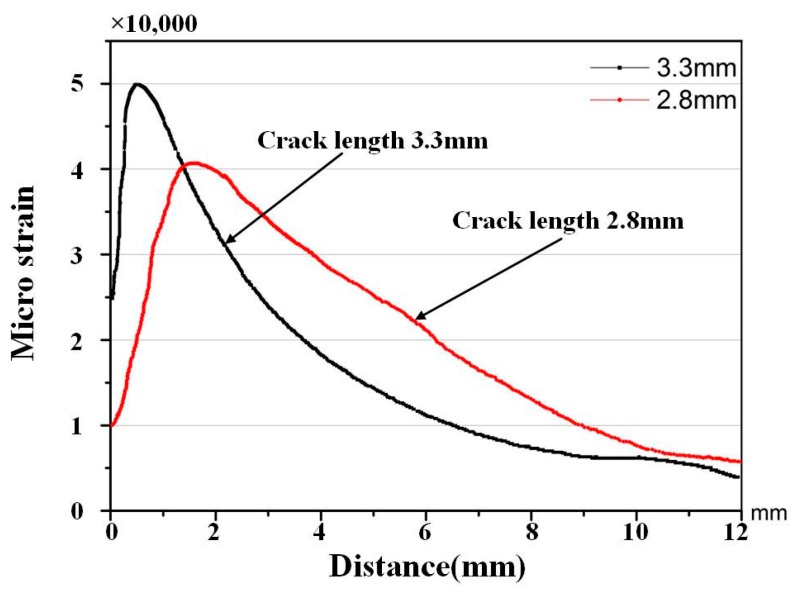
Strain value along the grating with crack lengths of 2.8 and 3.3 mm.

**Figure 13:**
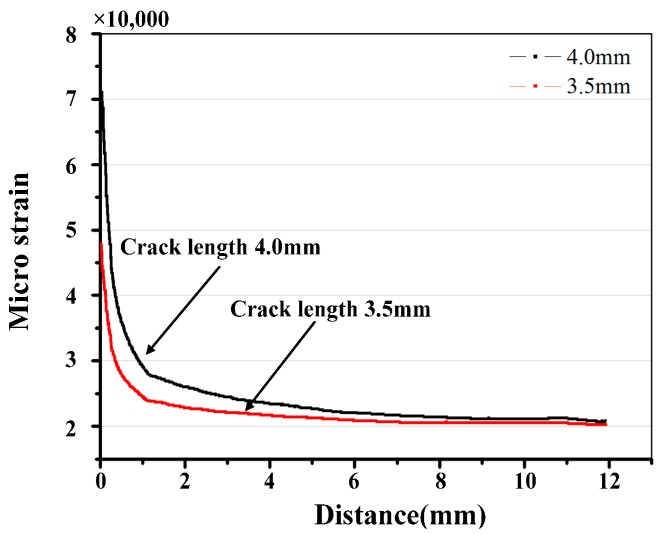
Strain value along the grating with crack lengths of 3.5 and 4.0 mm.

**Figure 14:**
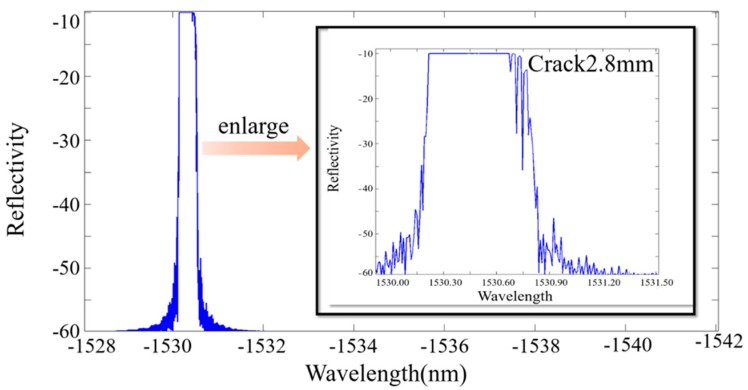
The simulated reflection intensity of a crack with a length of 2.8 mm.

**Figure 15:**
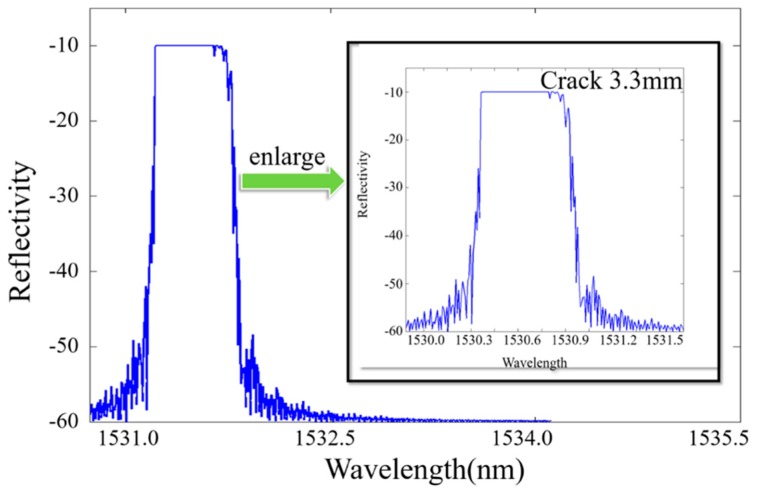
The simulated reflection intensity of a crack with a length of 3.3 mm.

**Figure 16:**
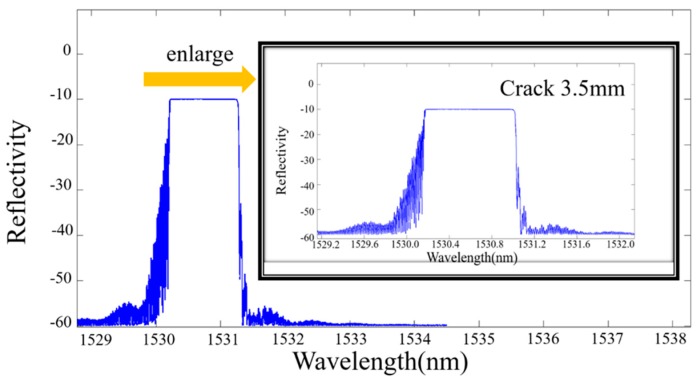
The simulated reflection intensity of a crack with a length of 3.5 mm.

The improved transfer matrix method (*T*-matrix method) was used to construct the reflection intensity spectrum of the FBG from the simulation profile along the grating of the FBG. The strain distributions along the grating at different cracks have been calculated in Section 4.1, as shown in Figure 12 and Figure 13. The simulation analysis was as follows: the main strains sensed by the adhered FBG were axial and lateral strains, but was more sensitive to axial strain. The FBG sensor profiles depend on the axial strain distribution ε(x) , in which x denotes the axial direction of the FBG, as shown in Equation (1):

(1) $$\Lambda \left(x\right)=\left(1+\varepsilon \left(x\right)\right)\Lambda_{ini},$$

wherein, $\Lambda_{ini}\;$is the initial grating period, Equation (1) expresses the geometrical elongation of a grating.

(2) $$n_{eff}\left(x\right)=n_{0}+\Delta n\left(x\right)=n_{0}-\frac{n_{0}^{3}}{2}\left\{p_{12}-v_{f}\left(p_{11}+p_{12}\right)\right\}\varepsilon \left(x\right),$$

wherein, $n_{0}$ is the initial refractive index of the core, $v_{f}$ is the Poisson’s ratio of the glass, and $p_{11}$, $p_{12}$ denote Pockel’s constants where indices 1 and 2 indicate the axial and transverse directions of the FBG. Equation (2) represents the change in the refractive index by the photo-elastic effect. The reflection spectra are obtained, as shown in Figure 14, Figure 15 and Figure 16.

### 4.3. Mechanism on the Various Location of the Subordinate Peak

In general, when the tensile strain is applied to the FBG, the reflected spectrum shifts to a higher wavelength, whereas when the compression strain is applied to the FBG, the reflected spectrum shifts to a lower wavelength [18]. The strain distribution at the crack tip area of the cracked specimen is highly heterogeneous, thus, with non-uniform strain distributed along the axis of the grating, the spectrum becomes broader, the reflectivity decreases, and the subordinate peak appeared initially, and even spectrum oscillation occurred.

Due to the local elastic-plastic material behavior, Ellyin [19] recognized the three different regions in the crack tip area, namely the monotonic plastic zone, cyclic plastic zone, and process zone. The stress ratio, $R_{\delta }$ in the crack tip region is decreased gradually from the process zone to the monotonic plastic zone. Kara [8] developed an experimental calibration of non-homogenous strain distribution along the grating. It reveals that a cubic strain distribution can be sensed by FBG.

As discussed in the above section, due to the complex plastic deformation ahead of the crack tip, a significant strain gradient is sensed by the grating of FBG sensors, and a quadratic strain distribution is employed along the grating of the FBG in the monotonic plastic zone and cyclic plastic zone ahead of the crack tip. By analysis of the collective data from the experiment, the subordinate peaks appeared in the longer wavelength of the reflection intensity spectrum. Meanwhile, the simulation of the reconstructed reflection intensity spectrum is approved from the FEM with the *T*-matrix method.

### 4.4. Analysis of Spectrum Oscillation

For metallic materials, the strain perceived by the FBG adhered to the surface is elastic strain $\varepsilon_{E}$, plastic strain $\varepsilon_{p}$, thermal strain $\varepsilon_{T}$, creep $\varepsilon_{\varphi }$, and $\varepsilon_{other}$, in which plastic deformation and elastic deformation are mainly perceived. The primary strain perceived by the FBG included elastic strain $\varepsilon_{E}$ and plastic strain $\varepsilon_{p}$. Meanwhile, the component of the strain distribution in the plastic strain $\varepsilon_{p}$, particularly in the process plastic zone ahead of the crack tip is complicated. This means the strain distribution along the grating is complex, including cubic, quadratic, and linear strain distribution along the grating when the crack propagates to the FBG. This is the reason why the oscillation occurs.

Furthermore, the strain distribution is highly heterogeneous in the process zone ahead of the crack tip; thus, the cubic strain component plays a significant role in the sensing of the FBG and the subordinate peaks appeared in the lower wavelength.

## 5. Conclusions

The experimental data from the fatigue testing is used to analyze the characteristic parameters for different crack lengths, combining with the various plastic zones. The spectrum became deformed with the crack propagation, such as the number of subordinate peaks, and the various locations of the subordinate wavelength.

The subordinate peak appeared at the higher wavelength, when the monotonic and cyclic plastic zones ahead of the crack tip approach the FBG sensor, and the strain distribution along the grating in these areas are quadratic. However, then the subordinate peak appeared at the lower wavelength, when the process plastic zone ahead of the crack tip approached the FBG sensor, and the strain distribution along the grating in this zone is cubic. In addition, with the complex plastic deformation in the process plastic zone, spectrum oscillation occurs when the crack reaches the FBG. Meanwhile, the FEM method is employed to extract the strain field at the crack zone. Based on the extractive strain field, the *T*-matrix method is used to construct the reflection intensity of the FBG sensor for different crack lengths. The subordinate peak shift, calculated from the reflection intensity spectra, is identified as a damage sensitivity index. In addition, the FBG optical spectrum oscillation occurring in the crack approaching the grating distribution was highly heterogeneous. The described crack propagation detection system may be applied in structural health monitoring.

## Figures and Tables

**Table 1:** Mechanical properties of 7075-T6 aluminum alloy plates.

Material	Elastic Modulus (MPa)	Poisson’s Ratio (MPa)	Yield Strength (MPa)	Tensile Strength (MPa)
AL7075-T6	73,100	0.33	503	572

**Table 2:** Properties of FBG sensors used for fatigue testing.

Parameter	FBG Sensor
Modulation mode of refractive index	Cosine
Effective refractive index $n_{eff0}$	1.460
Poisson’s ratio v	0.17
Grating length L (mm)	10.1
Center wavelength λ (nm)	1529.11
Photo-elastic coefficient $p_{11}$	0.113
Photo-elastic coefficient $p_{12}$	0.252
